# Isoniazid concentrations in hair and plasma area-under-the-curve exposure among children with tuberculosis

**DOI:** 10.1371/journal.pone.0189101

**Published:** 2017-12-07

**Authors:** Vidya Mave, Aarti Kinikar, Anju Kagal, Smita Nimkar, Hari Koli, Sultanat Khwaja, Renu Bharadwaj, Roy Gerona, Anita Wen, Geetha Ramachandran, Hemanth Kumar, Peter Bacchetti, Kelly E. Dooley, Nikhil Gupte, Amita Gupta, Monica Gandhi

**Affiliations:** 1 Byramjee-Jeejeebhoy Government Medical College-Johns Hopkins University Clinical Research Site, Pune, India; 2 Johns Hopkins University School of Medicine, Baltimore, Maryland, United States of America; 3 Byramjee-Jeejeebhoy Government Medical College, Pune, India; 4 University of California San Francisco, San Francisco, United States of America; 5 National Institute of Research in Tuberculosis, Chennai, India; University of Cape Town, SOUTH AFRICA

## Abstract

We measured hair and plasma concentrations of isoniazid among sixteen children with tuberculosis who underwent personal or video-assisted directly observed therapy and thus had 100% adherence. This study therefore defined typical isoniazid exposure parameters after two months of treatment among fully-adherent patients in both hair and plasma (plasma area under the concentration-time curve, AUC, estimated using pharmacokinetic data collected 0, 2, 4, and 6 hours after drug administration). We found that INH levels in hair among highly-adherent individuals did not correlate well with plasma AUC or trough concentrations, suggesting that each measure may provide incremental and complementary information regarding drug exposure in the context of TB treatment.

## Background

Tuberculosis (TB) is the most common cause of morbidity and mortality from an infectious agent globally [1]. Children with TB constitute 10% of the total global burden of TB with an approximate 1 million cases occurring among children younger than 15 years [1]. Maintaining adequate adherence to anti-TB drugs for a minimum of 6-months for active TB is essential (but challenging in children, the group at highest risk of TB-related mortality) [2]. Furthermore, each of the drugs in the recommended standard anti-TB treatment (ATT) regimen -isoniazid (INH), rifampicin (RIF), ethambutol (EMB) and pyrazinamide (PZA)- has a specific role in sterilizing both semi-dormant and actively multiplying mycobacterial bacilli, required for successful TB treatment outcomes [3](3). Finally, for global TB control and to reach the target of zero deaths from TB [4], it is critical to optimize existing TB prophylaxis strategies, including INH preventive therapy (IPT), specifically for children < 5years of age, who are at high risk of developing TB after household or other closely-affiliated exposure to active TB [1,2]. Maintaining adequate exposure to IPT is also a critical feature to its durable success.

Optimal drug exposure to ATT and IPT ensure killing of *Mycobacterium tuberculosis* bacilli, but best practices to measure drug exposure to anti-TB drugs remain elusive [5]. Treatment adherence as measured by adherence questionnaires and pill counts can be subject to social desirability and recall biases. Moreover, self-report or pill counts cannot capture actual drug ingestion and drug exposure [6]. Drug exposure is often measured by single plasma drug levels, but these levels represent only a small window of exposure and exhibit high rates of intra-individual variability [7,8]. Furthermore, plasma drug measurement requires skilled phlebotomists, cold chain for storage and shipment, and sampling timed to dose, limiting the use of this measure in routine clinical practice. Exposure measures from intensive pharmacokinetics (PK) studies are even more difficult to perform in clinical practice. Dried blood spot (DBS) analysis, using fingerprick sampling, is relatively simple, but this technology is still in the initial stages of development for anti-TB drugs and requires validation; moreover, the stability of some TB drugs (rifampin, isoniazid) in DBS is poor [9,10].

Hair assays of drugs have emerged as an alternate, noninvasive, innovative tool that can capture drug exposure and adherence [11]). Drugs gets incorporated into hair over weeks to months and concentrations in 1cm of hair represent approximately one month of drug exposure [12]. Our group has developed expertise in the development of hair assays for antiretrovirals (ARVs) and shown multiple uses for hair ARV concentrations in the setting of HIV treatment and prevention [13–19]. Building on earlier work by Eisenhut et al [20], we have previously shown that INH can be detected in the hair of children on ATT and that INH concentrations in hair are quantifiable over a dynamic range [21, 22]. Typical ranges of INH hair concentrations in fully adherent pediatric patients have not yet been defined. In this study, we provide initial data about typical values for INH hair levels and plasma area-under-the-curve concentrations from intensive PK studies- as well as their correlation- in a cohort of HIV-infected and uninfected children on ATT under directly observed therapy.

## Methods

A prospective cohort study of children with TB was established at BJ Government Medical College (BJGMC)—Sassoon General Hospital (SGH) in Pune, India. The details of the study are described elsewhere [22]. Briefly, we enrolled children <12 years with known HIV status who were initiated on first line thrice weekly ATT following a clinically or microbiologically confirmed TB diagnosis [22]. Of these, 16 participants underwent directly observed therapy (DOT) either using in-person DOT or using video-assisted DOT to ensure 100% adherence. No participant reported vomiting or diarrhea on the day of study measurements, and all had dark brown or black hair.

For this study, semi-intensive PK studies of INH were performed by obtaining plasma samples at 0, 2, 4, and 6 hours after directly-observed ATT administration at month 2 of treatment. Plasma concentrations of INH were measured using validated high performance liquid chromatography at the National Institute for Research in Tuberculosis (NIRT), Chennai, India [23]. On the same day of the plasma PK study and also at months 4 and 6, a small thatch of hair (approximately 20 strands) was cut from the occipital region close to the scalp and from underneath the top layer of hair as previously described [9,12]. The cut hair was then placed on a piece of tin foil and taped down at its distal end (side furthest from the scalp) with a thin label to mark directionality. The specimen was then sealed inside a plastic bag containing a desiccant pellet, stored at room temperature and shipped to the University of California, San Francisco, TB Hair Analysis Laboratory for measurement of INH concentrations, as previously described [22].

The median and interquartile ranges (IQR) of INH concentrations in hair were calculated. Similarly, median and IQR of plasma concentrations (maximum concentration, or C_max_) and exposure (area under the concentration-time curve over 6 hours (AUC_0-6_)) were calculated. The linear trapezoidal rule was used to compute AUC_0-6_; C_max_ was determined by visual inspection of data. Scatterplots were generated to compare log-transformed plasma AUC with hair concentrations. We examined the associations of plasma AUC at month 2 with hair INH levels at months 2, 4, and 6. Further, we compared plasma trough concentrations and the hair INH levels at month 2 and mean hair levels from month 2, 4 and 6 using Spearman rank correlation. The Byramjii-Jeejeebhoy Government Medical College Institutional Ethics Committee and Johns Hopkins University institutional review boards both approved the study. Parents/guardians provided written informed consent; participants, when appropriate, provided assent.

## Results

Of 16 children enrolled, the median age was 90 (IQR, 60–105) months; 5 (31%) were between 2 and <5 years of age and 11 (69%) were ≥5 years of age. Nine (56%) were female, 3 (19%) had HIV co-infection. The median weight was 19.5 (12.35–24.95) kg, and 9 (56%) reported a history of TB exposure within the last 2 years prior to enrollment; 8 (50%) had pulmonary TB and 8 (50%) were diagnosed with extrapulmonary TB (EPTB). In-person or video-recorded directly observed therapy verified 100% adherence to ATT for all 16 children.

The PK parameters from all children are shown in the **Table 1**. The median and IQR of INH concentrations in hair for months 2, 4 and 6 were 1.15 (0.54–2.25), 1.86 (0.81–6.32) and 0.90 (0.73–1.09) ng/mg, respectively. The Wilcoxon signed-rank test gave p > 0.95 for month 2 vs 4, p = 0.05 for month 2 vs 6, and p = 0.68 for month 4 vs 6. At 2 months, males tended to have higher INH hair values.

**Table 1:** Plasma area-under-the curve (AUC) concentrations and hair concentrations of isoniazid (INH) among children with 100% adherence to tuberculosis (TB) treatment compared to females (1.64 (1.21–13.2) vs. 0.66 (0.47–1.08), p = 0.05); INH hair levels were lower in older children (correlation = -0.42; p = 0.11) and among children who were heavier (-0.43; p = 0.10).

	Age in months	Sex	Weight in kgs	HIV Positive?	Plasma AUC INH levels, mcg/ml.hrs	Hair INH levels at month 2, ng/mg
**Child 1**	31	M	10	Negative	20.19	39.80
**Child 2**	113	F	24.9	Negative	23.3775	1.24
**Child 3**	60	F	12.7	Negative	33.225	1.08
**Child 4**	26	M	8	Positive	23.7825	13.2
**Child 5**	102	M	25	Negative	14.9850	1.601
**Child 6**	115	F	26	Negative	13.7175	0.823
**Child 7**	31	F	11	Negative	31.2525	19.5
**Child 8**	68	F	20	Negative	7.9875	0.205
**Child 9**	60	F	12	Positive	15.72	0.664
**Child 10**	104	F	19	Negative	22.425	0.466
**Child 11**	87	M	15	Negative	19.7925	2.858
**Child 12**	100	F	21	Negative	26.2125	0.103
**Child 13**	106	M	25	Positive	15.4425	1.635
**Child 14**	124	M	27	Negative	34.9275	0.365
**Child 15**	93	M	24	Negative	30.705	1.211
**Child 16**	78	F	19	Negative	5.5275	0.613

Kgs = kilograms, HIV = Human immunodeficiency virus

**Fig 1:**
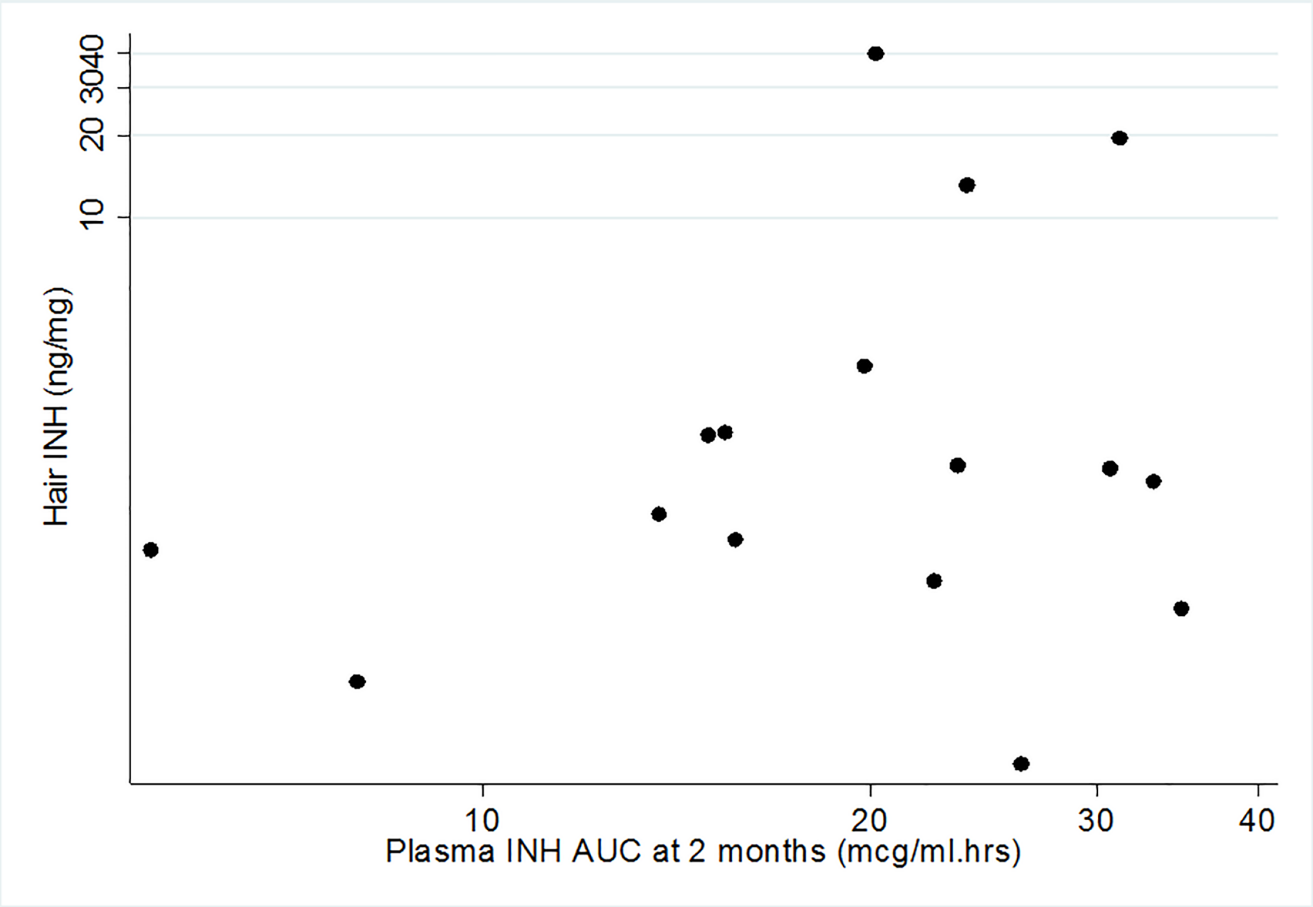
Scatterplot showing 6-hour plasma area-under-the-curve isoniazid concentrations and hair isoniazid concentration (log scale) at month 2 (n = 16).

The median (IQR) AUC was 21.31 (15.21–28.46) mcg/ml.hrs. In contrast to hair levels, median plasma AUC was similar in females (22.4 (13.7–26.2) mcg/ml.hrs) and males (20.2 (15.4–30.7) mcg/ml.hrs)p = 0.63. The **Fig** 1 shows the scatterplot of individual hair INH levels and corresponding plasma AUC measures of INH at month 2. Plasma AUC concentrations correlated poorly with hair INH concentration at month 2 (correlation coefficient 0.09 (-0.45, 0.62)); correlations were still poor, but improved at month 4 (correlation coefficient 0.21, 95% confidence interval [CI] (-0.42, 0.84)) and month 6 (correlation coefficient 0.38 (95% CI, -0.34, 1.00). Among males, the AUC-Hair correlation at month 2 was-0.32 (95% CI, -0.86, 0.56), while among females, it was 0.43 (95% CI, -0.33, 0.85). Plasma trough and peak levels correlated extremely poorly with hair INH levels with correlation coefficients of -0.060 (95% CI, -0.56, 0.44) and -0.25 (95%CI, -0.96, 0.47), respectively.

## Discussion

This study provides the first information about concurrently measured plasma and hair INH exposures among children with 100% adherence to ATT, as confirmed via in-person or video-assessed directly observed therapy. We found that hair levels of INH among highly-adherent individuals had little correlation with plasma AUC, peak or trough values, with plasma collected on a single occasion using semi-intensive PK sampling.

Suboptimal TB drug exposure increases the risk of TB treatment failure, resistance, recurrence and death, especially among children and HIV-infected persons [24–28]. While previous reports have demonstrated that inadequate INH exposure contributes to poor TB treatment outcomes in high HIV burden settings [25], limited data are available on target plasma drug concentrations associated with optimal TB treatment outcomes. A commonly-used ‘target’ C_max_ for INH is 3 mcg/mL, but this number is based on typical exposures seen in patients on TB treatment and not on convincing pharmacokinetic-pharmacodynamic (PK-PD) analyses. Single plasma levels, like C_max_, may be subject to significant day-to-day variation, with AUC measures from intensive or semi-intensive PK studies serving as more robust measures of exposure. However, calculating AUC parameters from even semi-intensive PK studies is an arduous process requiring a prolonged period of observation and multiple blood draws. Moreover, drug concentrations in hair provide an estimate of longer term exposure than a single AUC measure, for about one month of drug-taking if the hair is cut to 1 centimeter from the scalp [19]. Hair is feasible to collect, since it does not require phlebotomy, biohazardous precautions or a cold-chain, and we have shown high acceptability of hair collection in studies among Indian children [22](2).

Among children with TB, where drug taking relies on administration by the parent/guardian, and where drug levels can be highly variable due to maturing metabolizing systems, therapeutic monitoring of drug levels is likely to be critical for optimizing TB treatment outcomes. Given the noninvasive nature of hair sampling and the desire to avoid phlebotomy among young children, hair levels may provide a reasonable alternative to intensive PK sampling to assess drug exposure during ATT. This study showed poor correlation between hair levels of INH and plasma PK measures from a single time point. This lack of correlation may reflect the fact that hair assesses exposure over a much longer time frame than either a single plasma measure or a 6-hour AUC measure. Also, hair concentrations are influenced by both individual PK and by adherence over time, whereas single-occasion PK sampling simply demonstrates individual PK (which may depend on age, pharmacogenetics and other factors). Therefore, the measures may provide complementary information when assessing exposure among children on ATT, in the same way that a hemoglobin A1C and short-term blood glucose value may provide different but complementary information for a patient with diabetes. AUC measurement was only performed on one occasion and only over 6 hours, compared to the 48-72-hour inter-dose intervals, so it may not accurately measure the full drug exposure that contributes to hair levels. Furthermore, similar to data by Eisenhut et al [20], we found gender differences in hair INH levels, although these did not appear to explain the poor correlation of AUC and hair levels. Finally, it is possible that INH acetylator status may have influenced both hair and plasma drug levels as previously reported [20].

A major limitation of this study is that we used a very small subset of children from a larger study. Of note, as seen in other studies, there were a few hair and plasma levels that were extremely low. Since adherence was monitored and demonstrated to be 100%, the assumption is that extremely low levels reflect inadequate drug exposure due to pharmacokinetic variability (low absorption, rapid metabolism or clearance).

Pharmacologic measures are increasingly being explored to ensure efficacy of prophylactic and therapeutic regimens in both clinical studies and routine practice. Our study shows that hair levels provide information in highly-adherent patients that is distinct from the information provided by single-occasion exposure measures. Further study is required to assess the pharmacodynamic relationship between hair levels of TB drugs and treatment or prevention outcomes in large cohorts and the incremental value of adding hair to plasma exposure measures in these studies.
